# MicroRNA-27a Modulates HCV Infection in Differentiated Hepatocyte-Like Cells from Adipose Tissue-Derived Mesenchymal Stem Cells

**DOI:** 10.1371/journal.pone.0091958

**Published:** 2014-05-13

**Authors:** Jung Eun Choi, Wonhee Hur, Jung-Hee Kim, Tian Zhu Li, Eun Byul Lee, Sung Won Lee, Wonseok Kang, Eui-Cheol Shin, Takaji Wakita, Seung Kew Yoon

**Affiliations:** 1 The Catholic University Liver Research Center & WHO Collaborating Center of Viral Hepatitis, the Catholic University of Korea, Seoul, Republic of Korea; 2 Laboratory of Immunology and Infectious Diseases, Graduate School of Medical Science and Engineering, KAIST, Daejeon, Republic of Korea; 3 Department of Virology II, National Institute of Infectious Disease, Tokyo, Japan; Tulane University School of Medicine, United States of America

## Abstract

**Background and Aims:**

Despite the discovery of hepatitis C virus (HCV) entry factor, the mechanism by which it is regulated by miRNAs remains unclear. Adipose tissue-derived human mesenchymal stem cells (AT-hMSCs) have been widely used for differentiated hepatocyte-like cells (DHCs). Here, we established an *in vitro* HCV infection model using DHCs from AT-hMSCs and identified miRNAs that modulate HCV infectivity.

**Methods:**

AT-hMSCs were differentiated into DHCs using the conditional media, and evaluated for hepatocyte characteristics using RT-PCR, immunocytochemistry, periodic acid-Schiff staining, and a urea synthesis assay. The expression of HCV candidate receptors was also verified using immunocytochemistry. The levels of candidate miRNAs targeting HCV receptors were then determined by relative quantitative RT-PCR (rqRT-PCR). Finally, DHCs were infected using HCVcc and serum from HCV-infected patients, and infectivity of the virus was measured by rqRT-PCR and transmission electron microscopy (TEM).

**Results:**

The expected changes in morphology, function and hepatic gene expression were observed during hepatic differentiation. Moreover, the expression of candidate HCV entry factors and miR-27a were altered during hepatic differentiation. The infection and replication of HCV occurred efficiently in DHCs treated with HCVcc or infected with serum from HCV-infected patients. In addition, HCV infectivity was suppressed in miR-27a-transfected DHCs, due to the inhibition of LDLR expression by miR-27a.

**Conclusions:**

Our results demonstrate that AT-hMSCs are a good source of DHCs, which are suitable for in vitro cultivation of HCV. Furthermore, these results suggest that miR-27a modulates HCV infectivity by regulating LDLR expression.

## Introduction

Hepatitis C virus (HCV), a member of the *Flaviviridae* family, is a single-stranded positive-sense RNA virus with a viral genome length of approximately 9.6 kb in length. An estimated 180 million people worldwide are infected with HCV of which 55–85% may progress to chronic infection [1]. The standard therapy used to treat patients with chronic hepatitis C (CHC) is pegylated interferon in combination with ribavirin, but sustained virological response rates for specific HCV genotypes such as 1 or 3 remain low [2]. New antiviral agents including direct-acting agents (DAA) have therefore been extensively explored and are undergoing clinical trials. However, HCV research has been hampered by the lack of adequate viral culture system. Despite the ability of HCV infection in human primary hepatocyte [3] and immortalized human hepatoma cell lines [4], HCV replication remains inefficient and reproducibility has been poor. Previously, Wakita T *et al.* [5] established a culture system to robustly produce infectious HCV using genotype 2a (JFH1 strain) in human hepatoma (Huh7) cells. However, limiting the number of HCV variants from the broad range of genotypes that can be applied to this model. Recently, studies aimed at establishing an HCV *in vitro* infection system using differentiated hepatocyte-like cells (DHCs) were conducted. HCV pseudotype virus [6] and HCV derived from cell-culture (HCVcc, JFH1 strain) [7]–[9] were both used to confirm HCV infection in DHCs derived from induced pluripotent stem (iPS) cells. In addition, Wu *et al.* [10] suggested that DHCs derived from embryonic stem (ES) cells could be persistently infected with both HCVcc and HCV serum. Importantly, critical cellular cofactors that are required for HCV replication such as epidermal growth factor receptor (EGFR), ephrin receptor A2 (EphA2), phosphatidylinositol 4-kinase type III alpha (PI4KIIIα), and microRNA-122 are expressed in this model [11].

Despite efforts to establish an *in vitro* HCV infection model, it remains unclear whether cells differentiated from mesenchymal stem cells (MSCs) could become a reservoir for viral infection. MSCs are recognized as a more promising source than ES cells or iPS cells, which can trigger teratoma formation, immunogenicity, and are associated with ethical concerns [12], [13]. Currently, multipotent adipose tissue-derived MSCs (AT-hMSCs) can be differentiated into multiple cells, including adipocytes, osteoblasts, chondrocytes, myocytes, and hepatocytes [14], [15]. Although, bone marrow derived MSCs (BM-MSCs) are most widely used multi-potent MSCs [16], AT-hMSCs have a differentiation capacity comparable to that of BM-MSCs [17]. Moreover, AT-hMSCs are an attractive source of MSCs because larger quantities can easily obtained from cosmetic liposuction compared with bone marrow [18]. Our study therefore focused on establishing an *in vitro* HCV infection model using DHCs derived from AT-MSCs.

MicroRNAs (miRNAs) are a family of endogenous small non-coding RNAs, consisting of 20 to 25 nucleotides, that regulate gene expression at the post-transcriptional level in a sequence-specific manner [19]. Functionally, miRNAs regulate osteogenesis [20], chondrogenesis [21], adipogenesis [22], and hepatogenesis [23] by modulating gene regulatory networks. Previous studies revealed that several miRNAs such as let-7b [24], miR-27a [25], miR-122 [26], miR-155 [27], miR-196 [28], miR-199a [29], and miR-491 [30] regulate HCV replication. Specifically, the propagation of HCV was enhanced by liver-specific miR-122 in HCV non-permissive cells [31]–[33]. However, little is known about miRNAs that can make non-permissive cells susceptible to HCV infection.

We therefore established an *in vitro* HCV infection model using DHCs from AT-hMSCs which do not carry concerns over ethics, viral incorporation, or cancer development. We then assessed the ability of candidate miRNAs to regulate HCV entry receptors in an established HCV infection model.

## Materials and Methods

### Ethics statement

The study was conducted in accordance with the ethical guidelines and approved this study by Institutional Review Board of Seoul St. Mary's Hospital at the Catholic University of Korea. All patients, who provided their serums for this study, completed written informed consents before inclusion in the study. Also, their personal identifying information was restricted for analysis purpose and was not available to the public.

### Cell culture and hepatic cell differentiation

To differentiate MSCs into DHCs, AT-hMSCs were purchased from PromoCell (Heidelberg, Germany), and maintained in Mesenchymal Stem Cell Growth Medium (PromoCell). Huh7 and Huh7.5 cells were provided by Jane C. Moores (The Reagent of the University of California, Oakland, CA). The cells were maintained in Dulbecco's modified Eagle's medium (DMEM; Invitrogen, Carlsbad, CA) containing 10% FBS and 1% penicillin/streptomycin in a humidified incubator at 37°C with 5% CO_2_. For hepatic differentiation, passages 3–7 of AT-hMSCs were plated at 1×10^4^ cells/cm^2^ and incubated overnight. The cells were then washed with phosphate-buffered saline (PBS) and incubated in basal medium [60% DMEM-low glucose (Invitrogen), 40% MCDB201 (Sigma, St. Louis, MO), and 1% penicillin/streptomycin] with 20 ng/ml EGF (Peprotech, Rocky Hill, NJ) and 10 ng/ml bFGF (Peprotech) for 2 days. After incubation, hepatogenic cytokines and growth factors were sequentially added as follows: days 0–7 (step 1), basal medium with 20 ng/ml HGF (Peprotech) and 10 ng/ml bFGF; and days 7–21 (step 2), basal medium containing 20 ng/ml oncostatin M (Peprotech), 1 µmol/L dexamethasone (Sigma), and 50 mg/ml insulin-transferrin-selenium (ITS) premix (Invitrogen). The media were changed every 3 days.

### Reverse transcriptional-polymeric chain reaction (RT-PCR)

To detect the expression of mRNAs, complementary DNA (cDNA) was synthesized using RNA isolated from AT-hMSCs, DHCs, Huh7, and Huh7.5 cells based on the method of Hur *et al.* [34]. PCR was performed using specific primers (Table S1) and polymerase (Promega, Madison, WI) in a final volume of 25 µl. The number of cycles used is outlined in Table S1. The amplified products were separated on a 2% agarose gel containing 0.5 µg/ml ethidium bromide, and the nucleic acids were visualized under UV light using a Gel-Doc XQ system (Bio-Rad, Vienna, Austria).

### Flow cytometry analysis

To characterize the MSCs, the expression of known surface proteins from AT-hMSCs was assessed by flow cytometry using a fluorescence-activated cell sorter (FACS Vantage; BD BioSciences, San Diego, CA). The data were analyzed using Cell Quest Pro software (BD BioSciences). AT-hMSCs and DHCs were harvested and stained with FITC-conjugated anti-CD90 (1∶100; Milternyi Biotec, Auburn, CA) or FITC-conjugated anti-CD44 (1∶1000; BD BioSciences) antibodies at 4°C for 10 min. A minimum of 10,000 events were recorded and analyzed.

### Hepatic functional analysis

To assess hepatocyte function, glycogen production and urea synthesis were measured in the DHCs and AT-hMSCs. To evaluate glycogen production, cells were fixed using 4% paraformaldehyde for 30 min and incubated in 1% periodic acid (Sigma) for 10 min. The cells were then washed with PBS and incubated with Schiff's reagent (Sigma) for 15 min. The cells were then washed with tap water and mounted, and glycogen production was visualized by light microscopy (CK40; Olympus, Tokyo, Japan). To measure urea synthesis, cells were seeded at a density of 1×10^5^ cells per well in 6-well plates. The urea concentrations were then measured in the culture medium using a Quantichrom Urea Assay Kit (Bioassay System, Hayward, CA, USA) following the manufacturer's instructions.

### Immunocytochemistry (ICC)

AT-hMSCs and DHCs were fixed with Accustain (Sigma) or 4% paraformaldehyde for 30 min, and then rinsed three times (for 5 min each) with PBS. To inhibit the activity of endogenous peroxidase, cells were incubated with 3% hydrogen peroxide (Junsei Chemical, Kyoto, Japan) for 1 h, and then washed three times with PBS. After incubating for 1 h in blocking solution (1% goat serum in PBS), the cells were incubated for 2 h at 4°C with the following primary antibodies: mouse anti-albumin (DAKO, Glostrup, Denmark), rabbit anti-CD81 (BD Bioscience), rabbit anti-SR-B1 (Santa Cruz Biotechnology, Santa Cruz, CA), and rabbit anti-EGFR (Cell Signaling Technology, Danvers, MA). The cells were then washed three times with PBS, and the fluorescence signal was amplified using tyramide signal amplification (Invitrogen) following the manufacturer's instructions. To detect LDLR expression, cells were stained with 2 µg/ml Dil-LDL (Invitrogen) for 4 h at 37°C, and washed three times (for 5 min each) with PBS. The cells were then fixed for 30 min using 4% paraformaldehyde and rinsed three times with PBS for 5 min. Nuclei were visualized by staining for 5 min with 1 µg/ml 4,6-diamidino-2-phenylindol (DAPI, Sigma). After three washes, the Dil-LDL fluorescence intensity of the preparations was analyzed using a SpectraMax Gemini reader (Molecular Devices, Sunnyvale, CA). The preparations were mounted using Kalsers Glyceringelatine (Merck, Darmstad, Germany) and were examined by a fluorescence microscopy (Axiovert; Carl Zeiss, Gottingen, Germany).

### Transient transfection of miRNA mimics

The DHCs were seeded at a density of 1×10^5^ cells per well in 6-well plates, and transiently transfected with 20 nM miR-27a mimics (Genolution Pharmaceuticals, Seoul, Republic of Korea) or scrambled miRNA (miR-NC; Genolution Pharmaceuticals) by simultaneous seeding with G-fectin (Genolution Pharmaceuticals). The expression of LDLR was validated by Dil-LDL staining, which was carried out as described above.

### Relative quantitative real-time polymeric chain reaction (rqRT-PCR)

To quantify miRNA expression, RNA was isolated from AT-hMSCs and DHCs using TRIzol reagent (Invitrogen) following the manufacturer's instructions. miRNAs expression was then measured using the miRNA detection service from Genolution Pharmaceutics; each sample was normalized to the expression of U6 miRNA.

The levels of HCV RNA in HCV-infected cells were also assessed by rqRT-PCR. An HCV-specific probe, 5′-FAM-CGC AGA CCA CTA TGG CTC TCC CG-BHQ-1-3′ (Metabion, Martinsried, Germany), was synthesized. Amplification was carried out using the LightCycler 480 system (Roche Applied Science, Basel, Switzerland) with a PCR mixture containing 5 pmol of forward primer (5′-ATG GCG TTA GTA TGA GTG TCG T-3′), 5 pmol of reverse primer (5′-YGG GTT KAT CCA AGA AAG GAC-3′), and 5 pmol of HCV-specific TaqMan probe. The thermal conditions were designed using the Roche Universal Probe Library's thermocycling conditions following the manufacturer's instructions. Human β-actin was used as a reference gene. All fluorescence data were analyzed using the LightCycler 4.0 software (Roche Applied Science), and C_t_ results were exported to Excel sheets. The comparative C_t_ method was used for relative quantification and normalization.

### HCV infection

To infect DHCs with HCV, AT-hMSCs and DHCs were seeded at a density of 1×10^5^ cells per plate on 35-mm dishes 1 day prior to infection. The cells were infected using 1 MOI of HCVcc (generously provided by Dr. Eui-Cheol Shin, Korea Advanced Institute of Science and Technology, Daejeon, Republic of Korea), serum from a genotype-1 CHC patient (Seoul St. Mary's Hospital, Seoul, Republic of Korea), and serum from a genotype-2 CHC patient (Seoul St. Mary's Hospital). After 4 h, the cells were rinsed 6–10 times with PBS and 1.5 ml of fresh growth medium was added. The expression of HCV RNA was then analyzed by rqRT-PCR as described above.

### Transmission electron microscopy (TEM)

Seven days after infection, AT-hMSCs and DHCs were fixed overnight using 2% glutaraldehyde in 0.1 M sodium cacodylate at 4°C. The cells were then processed for TEM as described previously [35].

### Statistics analysis

All data are representative of a minimum of three independent experiments. Sigma Plot (Systat Software. Inc. San Joes, CA) was used to run paired Student's *t*-test. Difference were considered to be statically signifiant when *p*<0.05.

## Results

### Changes in morphology, function, and hepatic gene expression during the differentiation of AT-hMSCs into DHCs

**Figure 1:**
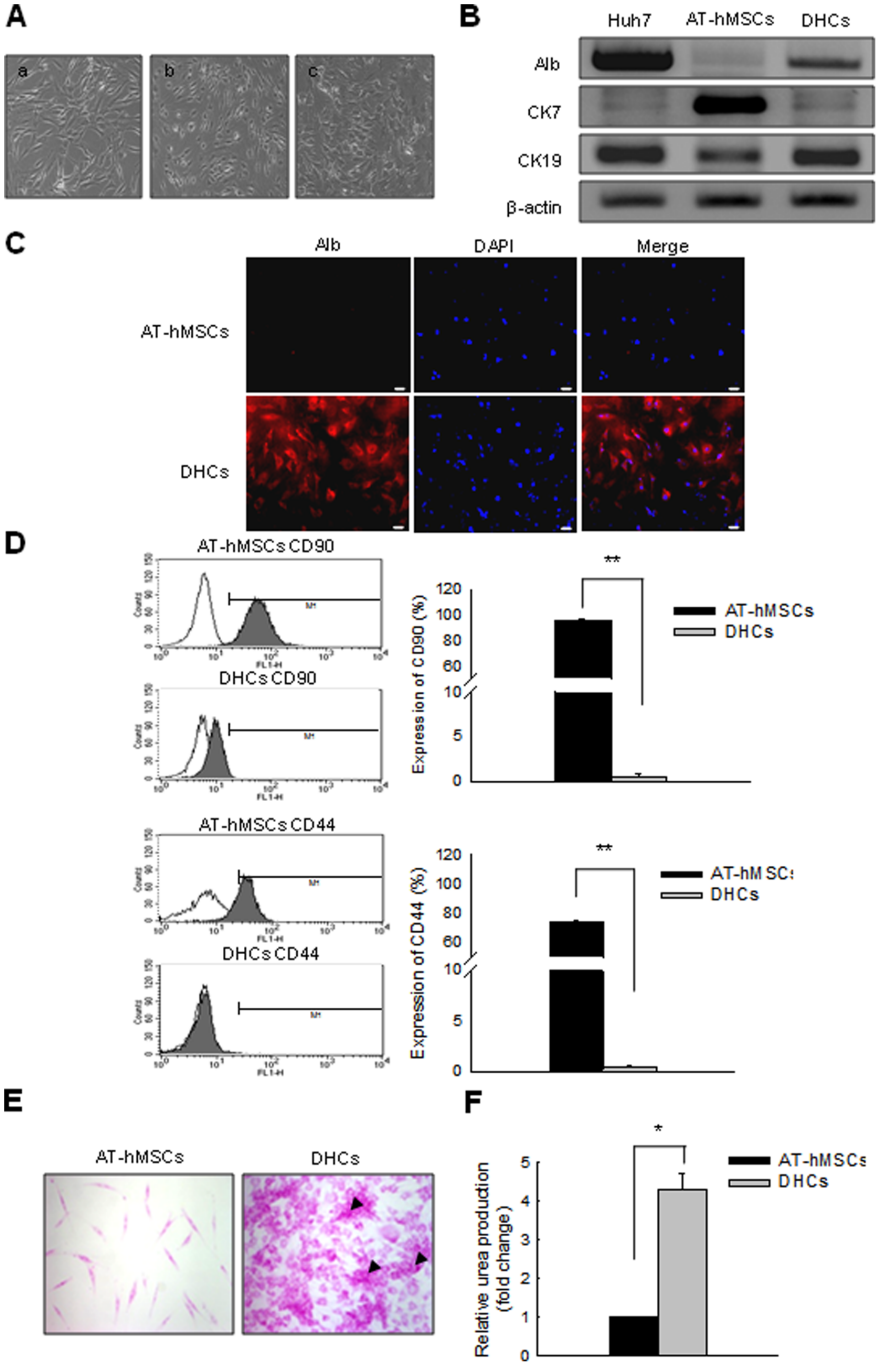
Successful differentiation of AT-hMSCs into DHCs in conditioned medium. AT-hMSCs were differentiated over 3 weeks using a two-step protocol. (A) The morphology of the cells changed markedly from a fibroblast-like morphology to a polygonal shape during differentiation. (a) Pre-treatment. (b) After treatment with one-step medium. (c) After treatment with two-step medium. To determine whether the cells exhibited increased in the expression of hepatic markers, we assessed the expression of hepatic markers at the RNA (B) and protein levels (C). (B) RT-PCR analysis for the detection of Alb, CK7, and CK19 expression in Huh7 cells, AT-hMSCs, and DHCs. β-Actin served as a loading control; Huh7 was the positive control. (C) Alb protein expression was detected in AT-hMSC and DHCs by ICC. These cells were stained with anti-albumin (red) and DAPI (blue), and image merging revealed strong cytoplasmic localization of Alb (indicated by the arrows) in DHCs. (D) To verify whether the DHCs exhibited decreased expression of AT-hMSC markers during differentiation, AT-hMSCs and DHCs were stained with FITC-conjugated CD90 or CD44. The FITC-positive cells were then examined by FACS analysis. To certify the hepatic function of the DHCs, PAS staining (E) and urea assays (F) were carried out using AT-hMSCs and DHCs. (E) Stored glycogen is represented by pink staining; arrows show cells with high levels of stored glycogen. (F) Urea production was measured in AT-hMSCs or DHCs. Scale bars are 50 µm. All data are representative of at least three independent experiments. *p<0.05 and **p<0.001 compared with the control.

To assess the capacity of AT-hMSCs to differentiate into hepatocytes, AT-hMSCs were differentiated using two-step conditioned media over 3 weeks. Morphological changes were observed in AT-hMSCs during differentiation (Fig. 1A). At the start, the AT-hMSCs exhibited a fibroblast-like morphology (Fig. 1A [a]). As the cells differentiated, they began to evolve into round or polygonal shapes (Fig. 1A [b] and 1A [c]). In addition, the expression of hepatocyte- and AT-hMSC-specific markers was examined in AT-hMSCs and DHCs. The mRNA expression of immature hepatocyte markers such as cytokeratin7 (CK7) was decreased in the DHCs compared with the AT-hMSCs (Fig. 1B). In contrast, the expression of mature hepatocyte markers such as albumin (Alb) and cytokeratin19 (CK19) was elevated in DHCs compared with AT-hMSCs (Fig. 1B). Importantly, expression of the AT-hMSCs markers such as CD90 and CD44 decreased significantly during differentiation. Specifically, FACS analysis showed that the number of CD90- and CD44-positive cells in the DHCs compared with the AT-hMSCs decreased from 96 to 0.6% (CD90), and 74 to 0.4% (CD44); *p*<0.001. Glycogen storage and urea synthesis in DHCs were also examined to evaluate the functional differentiation of the DHCs. Periodic acid-Schiff (PAS) staining revealed that the DHCs were capable of specific glycogen storage compared with AT-hMSCs (Fig. 1E). Furthermore, the urea synthesis ability of the DHCs was enhanced significantly by 4.26-fold compared with the AT-hMSCs (*p*<0.05, Fig. 1F). These results demonstrate that AT-hMSCs well differentiated into DHCs using two-step conditioned media.

### Expression of candidate HCV entry receptors in DHCs

**Figure 2:**
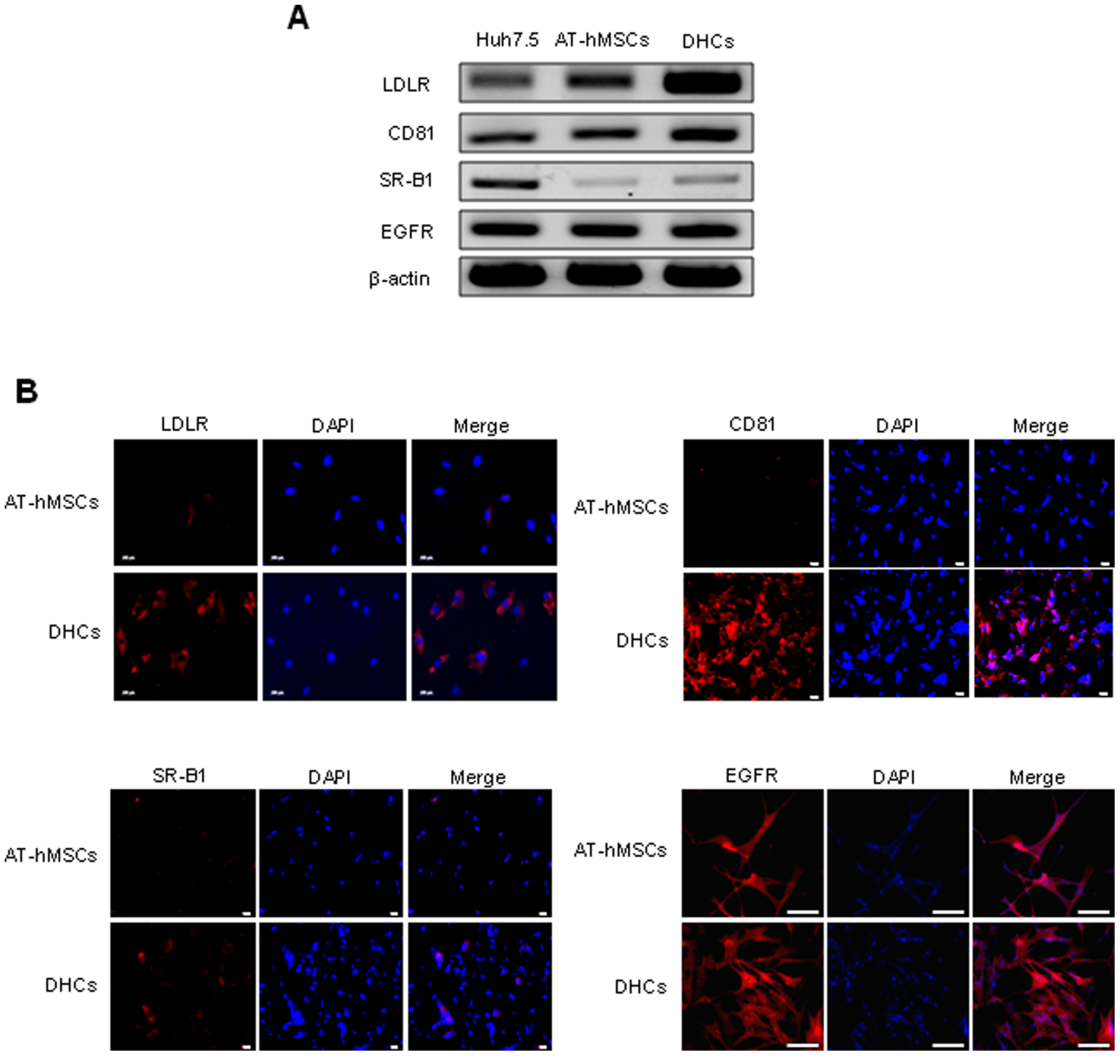
Expression of candidate HCV entry receptors in DHCs. To evaluate the possible routes of HCV infection, the mRNA (A) and protein (B) expression of various HCV candidate receptors was assessed in DHCs. (A) RT-PCR analysis of the level of LDLR, CD81, SR-B1, and EGFR in Huh7.5 cells, AT-hMSCs, and DHCs. β-Actin was used as the loading control; Huh7.5 served as the positive control. (B) To detect the protein expression of candidate HCV entry receptors, the cells were stained with Dil-LDL (red), anti-CD81 antibodies (red), anti-SR-B1 antibodies (red), or anti-EGFR antibodies (red), and DAPI (blue). Merging of the images revealed strong expression of candidate HCV entry receptors in the DHCs. Scale bars are each 100 µm (LDLR), 50 µm (CD81 and SR-B1), and 200 µm (EGFR).

Several candidate HCV entry receptors have been reported, including LDLR, CD81, SR-B1, and EGFR [36]. We therefore evaluated the expression of these candidate HCV entry receptors in DHCs to determine the susceptibility of DHCs to HCV infection. The mRNA and protein expression of the candidate HCV entry receptors are shown in Fig. 2A and B respectively. We observed increased expression of LDLR, CD81, and SR-B1 in DHCs compared with AT-hMSCs while the levels of EGFR were similar in the two cell types. This suggests that DHCs express potential HCV entry receptors.

### Susceptibility of DHCs to HCV infection

**Figure 3:**
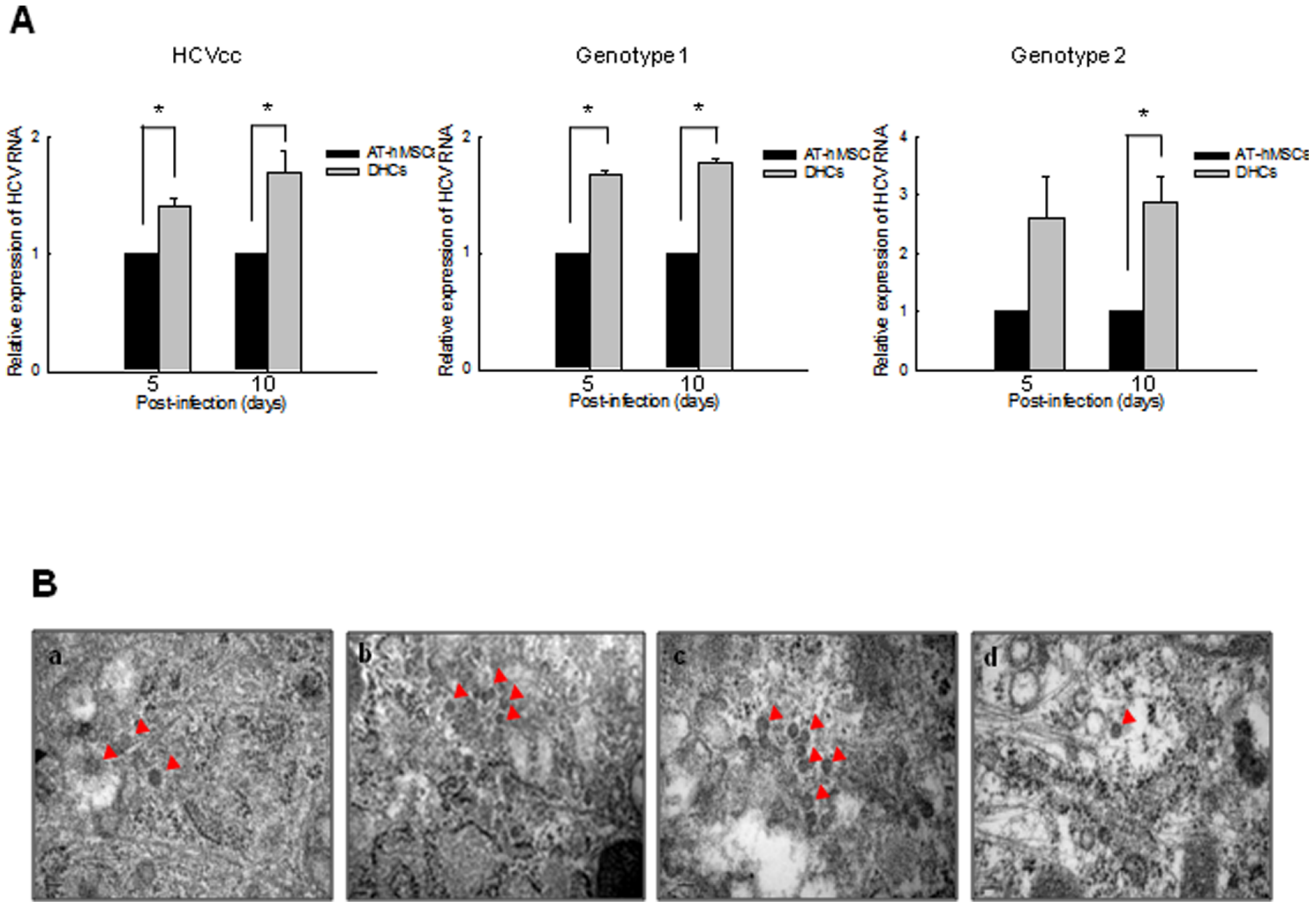
HCV infection assay using HCVcc or serum from CHC patients in DHCs. To determine whether HCV could infect DHCs, we infected cells with either HCVcc or serum from CHC-patients (genotype 1 or 2). (A) The relative expression of HCV RNA was assessed by rqRT-PCR in DHCs that had been infected with HCVcc, genotype 1 CHC-infected serum, or genotype 2 CHC-infected serum. (B) The presence of HCV particles was then detected using TEM in HCVcc (a), genotype 1 CHC serum- (b), and genotype 2 CHC serum-infected DHCs (c). (d) HCVcc-infected Huh7.5 cells were used as a positive control. Red arrows indicate HCV particles. Scale bars = 100 nm. All data are representative of at least three independent experiments. *p<0.05 compared with the control.

To confirm the replication and translation potential of HCV, AT-hMSCs or DHCs were infected with HCVcc or the serum of patients infected with HCV. The levels of HCV RNA were then measured 5 and 10 days post-infection, using rqRT-PCR. HCV RNA levels were significantly higher in HCVcc-infected DHCs compared with HCVcc-infected AT-hMSCs (*p*<0.05, Fig. 3A). Consistent with this, HCV RNA levels were increased significantly in DHCs infected with patient sera compared with AT-hMSCs, independent of the HCV genotype. In addition, expressions of HCV proteins NS5A and NS5B was detected in DHCs 10 days after HCV infection (data not shown). HCV particles, approximately 50 nm in diameter were also observed in the cytoplasm of HCV-infected DHCs using TEM (Fig. 3B), but not in HCV-infected AT-hMSCs (data not shown). These results demonstrate that DHCs infected with HCVcc or HCV patient sera are suitable as an *in vitro* HCV infection model.

### The expression of regulatory miRNAs is associated with candidate HCV entry receptors in DHCs

**Figure 4:**
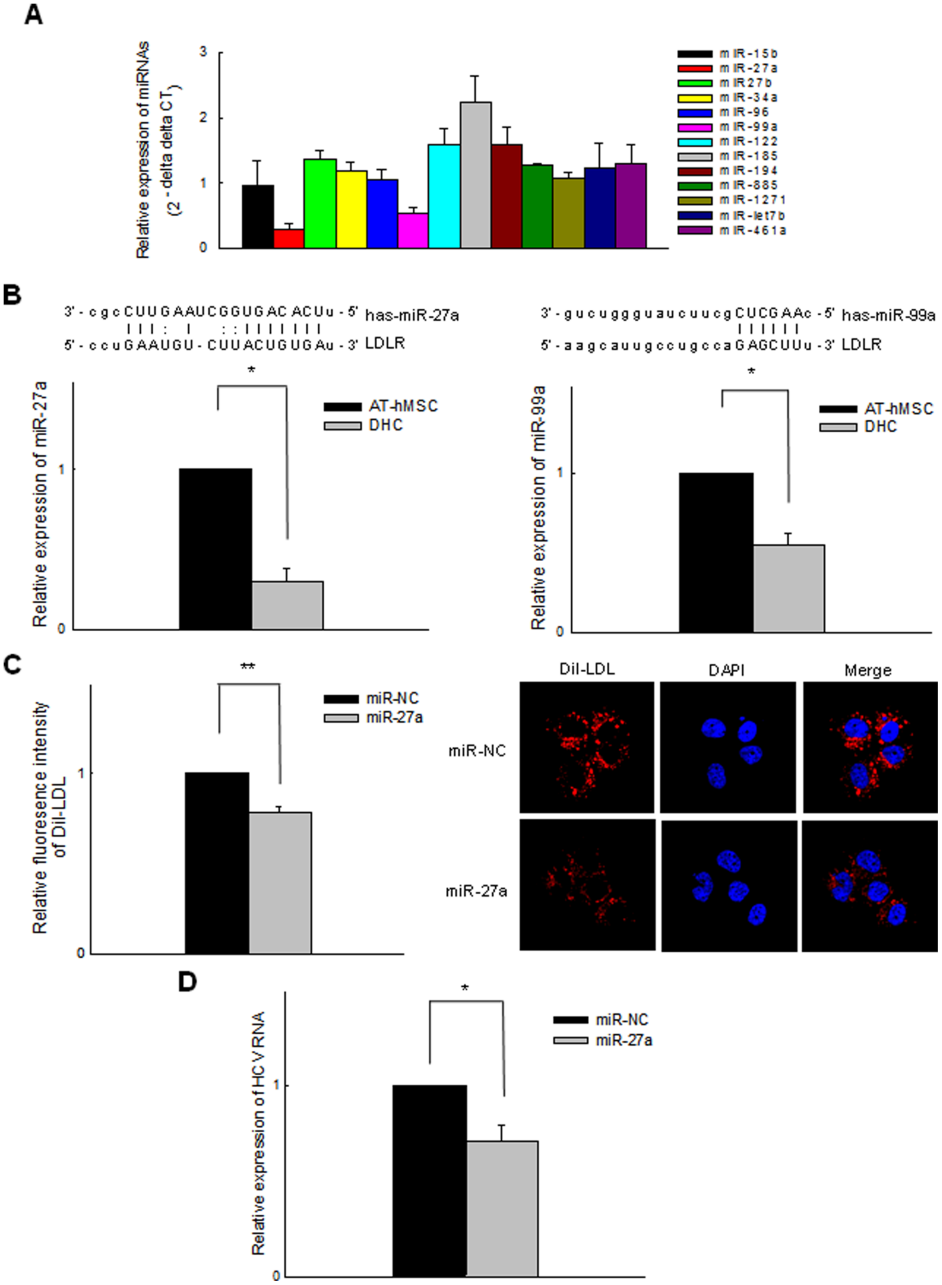
MiR-27a regulates HCV infectivity in DHCs. (A) To examine the expression of miRNAs that may target candidate HCV entry receptors, relative miRNA expression data were obtained using rqRT-PCR. The ΔCT method was used to calculate the relative expression of each miRNA (2^−[ΔCT mean of DHCs – ΔCT mean of AT-hMSC]^). (B) To identify the expression and target of miR-27a and -99a in DHCs, we anticipated the targets of the miRNAs using miRNA prediction software. Both miR-27a and -99a had a target site in the LDLR. In addition, differential expression of miR-27a and miR-99a was detected in AT-hMSCs and DHCs. Each sample was normalized to the expression of U6 miRNA. (C) To determine whether miR-27a targets LDLR, mimics of miR-27a were transfected into DHCs, and the relative expression of LDLR was calculated based on Dil-LDL staining. Each sample was normalized to the DAPI fluorescence intensity. Confocal microscopic images represent the fluorescence intensity of Dil-LDL (red) and DAPI (blue). Original magnification, 400×. (D) To verify that miR-27a regulates HCV infectivity, DHCs that had been transfected with miR-27a mimics were infected with HCV cc and rq-RT-PCR analysis was used to assess the relative expression of HCV RNA 5 days later. All data are representative of at least three independent experiments. *p<0.05 and **p<0.001 compared with the control.

Recent reports have described that miRNAs play an important role in the hepatic differentiation [37] and HCV entry [10]. Although Ishida *et al.* [30] revealed that miR-491 regulates HCV replication, the miRNAs associated with HCV entry were not completely identified. We therefore tried to find for candidate miRNAs associated with HCV entry using miRNA prediction software (http://www.microrna.org) and selected 13 candidate miRNAs. The putative targets of these miRNAs are listed in Table S2. We then measured the expression of these miRNAs during hepatic differentiation using rqRT-PCR. Six miRNAs (miR-27b, -122, -185, -194, -885, and -1271) were upregulated but miR-27a and -99a were down-regulated in DHCs compared with AT-hMSCs (Fig. 4A). In contrast, the expression of miR-15b, -34a, -96, -let7b, and -461a was comparable between DHCs and AT-hMSCs. We excluded the six upregulated miRNAs from further study because they are unlikely to be involved in HCV entry receptors expression during hepatic differentiation. The two miRNAs that were downregulated were further assessed to determine whether they affect the susceptibility of cells to HCV infection.

### MiR-27a regulates HCV infectivity by targeting LDLR in DHCs

The expression of miR-27a and -99a was significantly decreased in DHCs compared with AT-hMSCs (*p*<0.05, Fig. 4B), and so we identified downstream targets of these miRNAs. As shown in Table S2 LDLR was identified as a direct target of miR-27a and miR-99a. We therefore assessed the relationships between LDLR expression and the levels of miR-27a and -99a. As the expressions of miR-27a and miR-99a decreased during hepatic differentiation the expression of LDLR expression increased, suggesting that these miRNAs regulate LDLR expression. We next investigated which of the miRNAs directly regulates LDLR by transfecting DHCs with mimics of miR-27a and miR-99a and then staining them with Dil-LDL. The fluorescence intensity of Dil-LDL was decreased significantly in miR-27a-transfected DHCs compared with scrambled miRNA-transfected cells (*p*<0.001, Fig. 4C). In contrast, miR-99a had no effect on the expression of LDLR (data not shown). We therefore selected miR-27a to verify that the regulation of HCV infectivity occurs via LDLR expression. DHCs that had been transfected with miR-27a mimics were infected with HCVcc, and the expression of HCV RNA was quantified. The levels of HCV RNA in DHCs transfected with miR-27a mimics were reduced significantly compared with scrambled miRNA-transfected DHCs (*p*<0.05, Fig. 4D). These results suggest that the overexpression of miR-27a down-regulates LDLR expression, may lead to reducing HCV entry in an *in vitro* HCV infection model.

## Discussion

In the present study, we successfully developed an *in vitro* HCV infection model using DHCs derived from AT-hMSCs. Many types of stem cells, including ES cells, iPS cells, and MSCs, have been used for hepatic differentiation. Although ES cells are pluripotent and can efficiently differentiate into DHCs, they have limited biological applications because of concerns over teratoma formation, ethical issues, and immune rejection [12]. Although iPS cells can overcome the limitations of ES cells, they are not suitable for virus-related research because they are generated by permanent infection with viruses carrying several genes associated with reprogramming [38]. The integration of viral genomes containing reprogramming factors into host chromosomes could give unexpected results and make the data difficult to interpret. We therefore used the AT-hMSCs for hepatic differentiation to develop an *in vitro* HCV infection model. Recently, Banas *et al.* [15] showed that it takes 5 weeks to differentiate AT-hMSCs into functional DHCs. However, we obtained DHCs derived from AT-hMSCs within 3 weeks using our differentiation protocol. In spite of the reduced differentiation period, the differentiated AT-hMSCs obtained by this study were comparable to those described previously. During differentiation, the stem cell characters of AT-hMSCs were lost and hepatocyte properties were acquired. To evaluate the suitability of these cells as an *in vitro* HCV infection model, we investigated the expressions of HCV entry receptors, including LDLR, CD81, SR-B1, and EGFR. A recent study reported that ES cell-derived DHCs expressed CD81, claudin-1, claudin-6, LDLR, occludin, and SR-B1 at the mRNA level, and CD81 and SR-B1 at the protein level [7]. Consistent with this, we observed high levels of expression of LDLR, CD81, and SR-B1 suggesting that these factors regulate HCV infection in DHCs derived from AT-hMSCs. However, the expression of EGFR was already upregulated in AT-hMSCs and did not change during differentiation, suggesting that it plays little to no role in hepatic differentiation. This could be explained by previous observations, in which the expression of EGFR was elevated to induce self-renewal in MSCs [39].

Although the specific differentiation of MSCs into multiple lineages has been widely described, this is the first study to develop a reservoir for HCV using DHCs derived from AT-MSCs. Until now, MSCs or cells derived from MSCs were often used as the reservoir for viral infection. For example, undifferentiated BM-MSCs were susceptible to herpesvirus and cytomegalovirus, but the periods of replication were transient [40]. More recently, Nazari-Shafti *et al.* [41] showed that hematopoietic cells differentiated from AT-hMSCs were permissive to infection with human immunodeficiency virus type 1 (HIV-1), but undifferentiated cells were not. In this study, we observed that the susceptibility of DHCs to HCV is higher than in AT-hMSCs. In addition, DHCs could be infected with both HCVcc and patient-derived HCV inoculums, suggesting that they are susceptible to infect with different types of HCV. However, although HCV RNA levels could be measured by rq-RT-PCR in HCV-infected DHCs, the level of HCV proteins were too low to be visualized by western blotting. We therefore confirmed that HCV proteins were translated by presence of HCV particle in HCV-infected DHCs using TEM. These data demonstrate that HCV can enter into DHCs derived from AT-hMSCs, replicate its genome, and produce new viral particles in the cells.

This is the best knowledge the first report to show that miRNAs regulate HCV entry receptors. The mechanism of viral entry into cells involves envelope glycoproteins of HCV, which interact with cellular factors, including cell surface receptors and associated factors such as CD81, SR-B1, LDLR, claudin-1, occludin, CD209, CD209L, glycosaminoglycans, EGFR, CD5, and Niemann-Pick C1-like 1. Previous studies revealed that factors such as polarization and EWI-2 expression disrupt the cellular entry of HCV. More recently, Narbus *et al.* [33] demonstrated that miR-122 modulates the susceptibility of cells to HCV infection by showing that non-permissive HepG2 cells were successfully infected with HCV when treated with miR-122 mimics. However, they unable to identify the host factors associated with HCV entry that was modulated by miR-122. In this study, we found that miRNAs regulate the expression of HCV entry receptors, and investigated their role in HCV infection. We found that miR-27a and -99a were down-regulated during hepatic differentiation, and that their putative targets are EGFR and LDLR. Because the expression of EGFR did not change during hepatic differentiation, we focused on the relationship between miR-27a and LDLR expression in DHCs using miR-27a mimics. Previously, Shirasaki *et al.* [42] reported that miR-27a inhibited HCV replication in hepatoma cell lines by regulating lipid accumulation. However, they did not demonstrate a relationship between the expression of LDLR and miR-27a, and HCV. Here, we showed that treatment with miR-27a mimics resulted in suppressed LDLR expression, followed by reduced HCV infectivity, in DHCs. This suggests that the downregulation of miR-27a during hepatic differentiation makes DHCs more susceptible to HCV infection and additional inhibition of miR-27a expression might enhances the susceptibility to HCV infection. In further studies, it would be necessary to investigate the improvement of HCV in vitro infection model by modulation of miRNAs expression.

In conclusion, we established a suitable *in vitro* HCV infection model using DHCs from AT-hMSC with various HCV inoculums. We also demonstrated the expression of miRNAs and candidate HCV entry receptors during hepatic differentiation. Furthermore, we demonstrated that miR-27a modulates HCV infectivity by regulating the expression of candidate HCV entry receptors in an established *in vitro* HCV infection model. These findings are applicable to the identification of HCV entry factors and the vaccine development of HCV using an *in vivo* HCV infection model.

## Supporting Information

Table S1 List of primer sequences used for RT-PCR.
(DOCX)

Table S2 Putative target genes of up-regulated or down-regulated miRNAs.
(DOCX)
