# Supplementary material for: MicroRNA-27a Modulates HCV Infection in Differentiated Hepatocyte-Like Cells from Adipose Tissue-Derived Mesenchymal Stem Cells
Source: PLoS One. 2014 May 13;9(5):e91958. doi: 10.1371/journal.pone.0091958 (PMC4019502; doi:10.1371/journal.pone.0091958)
Supplement: Table S1 — List of primer sequences used for RT-PCR. (DOCX) [file pone.0091958.s001.docx]

**Supplementary Tables**

Table S1. List of primer sequences used for RT-PCR

| Name | Primer sequences | Annealing Temp. (˚C) | Cycles |
| --- | --- | --- | --- |
| Alb | F : 5’-CTG GCC TAC AAC AAG TTC TC-3’ | 55 | 35 |
|  | R : 5’-GAT ACT GGA TTC CTG GAA CA-3’ |  |  |
| CK7 | F : 5’-ATT CCA CTG GTG GCA GTA GC-3’ | 40 | 32 |
|  | R : 5’-GGG TGG GAA TC TCT TGT GA-3’ |  |  |
| CK19 | F : 5’-TTT GAG ACG GAA CAG GCT CT-3’ | 40 | 32 |
|  | R : 5’-AAT CCA CCT CCA CAC TGA CC-3’ |  |  |
| CD81 | F : 5’-AGA TCG CCA AGG ATG TGA AG-3’ | 50 | 36 |
|  | R : 5’-GGT TGC TGA TGA TGT TGC TG-3’ |  |  |
| SR-B1 | F : 5’-CTG TGG GTG AGA TCA TGT GG-3’’ | 55 | 36 |
|  | R : 5’-GCC AGA AGT CAA CCT TGC TG-3’ |  |  |
| LDLR | F : 5’-AGC TTG ACA GAG CCC ACG GCG-3’ | 50 | 38 |
|  | R : 5’-CGT TCT CCC TGT ATA TCG TTT-3’ |  |  |
| EGFR | F : 5’-CAG CGC TAC CTT GTC ATT CA-3’ | 50 | 38 |
|  | R : 5’-TGC ACT CAG AGA GCT CAG GA-3’ |  |  |
| β-actin | F : 5’-GGC ACC CAC CCT TCT ACA ATG A-3’ | 50 | 25 |
|  | R : 5’-CCC TCG TAG ATG GGC ACA CT-3’ |  |  |
