# Supplementary material for: MicroRNA-27a Modulates HCV Infection in Differentiated Hepatocyte-Like Cells from Adipose Tissue-Derived Mesenchymal Stem Cells
Source: PLoS One. 2014 May 13;9(5):e91958. doi: 10.1371/journal.pone.0091958 (PMC4019502; doi:10.1371/journal.pone.0091958)
Supplement: Table S2 — Putative target genes of up-regulated or down-regulated miRNAs. (DOCX) [file pone.0091958.s002.docx]

**Supplementary Tables**

Table S2.Putative target genes of up-regulated or down-regulated miRNAs

| Up-regulated miRNAs | Putative target |
| --- | --- |
| miR-27b | NOVA1, AKIRIN1, GCC2, HDHD2, ARX, PLK2, SBF2, USP42, FBXW7, CDH11, SNAP25, ALCAM, ZDHHC17, RNMT, , DUT, ADAM9, MBTD1, EYA4, SNRNP27, CACNA2D3, PLCH1, KDM3A, **LDLR**, ZNF542, RREB1, PXDN, GALNT7, ZNF781, KIAA0146, YWHAQ, NRK, UBE2F, RGPD5, **EGFR**, GRIA4, PAX9, RGPD6, SFXN2, RPS6KA5, MIER3, RGPD4, MAP2K4, PDE7B, **SCARB1,** … |
| miR-122 | RIMS1, C17orf104, C10orf118, LMNB2, CLIC4, STAG3L1, RNF32, DYT3, COLEC12, RFXAP, SLC9A6, ACER2, GALNT3, TMEM87A, SLC9A1, MAP3K12, ANGEL2, BCL2A1, CUX1, **LDLR**, MYO9A, CCAR1, CD40LG, EMR4P, GABRE, SHPRH, C15orf53, BPY2, DKFZP434H168, **CD81**, TMX3, CCDC127, YWHAQ, TAPT1, GALNT12, STAU1, NPEPPS, FADS1, CTTNBP2NL, … |
| miR-185 | PCDHA12, PCDHA11, PCDHA10, PCDHAC2, PCDHAC1, PCDHA13, PCDHA5, PCDHA6, PCDHA7, PCDHA8, PCDHA2, PCDHA1, PCDHA4, PCDHA3, SET, MLLT11, NPL, DGKB, **LDLR**, **CD81**, TUBGCP4, SIX2, CAPZB, FCRL3, CDC20B, C6orf134, **EGFR**, CNTD1, ABCG4, MYST3, CXCL9, SLC17A7, SF1, **SCARB1**, SMG7, VEGFA, BASP1, CA10, AKR1C1, KPRP, PM20D1, AKR1C2, LZTFL1, MDM4, GPR84, AGER, SLC16A2, LOC153469, KRTAP5-4, … |
| miR-194 | FLJ43390, EPC2, PTPN20A, PTPN20B, NEUROD1, CHORDC1, SLK, HBEGF, FMR1, TMBIM4, QKI, DAZ2, RWDD4A, ADAM10, **CD81**, JARID2, SLC39A6, **EGFR**, GORAB, PAIP2, KIAA1239, LRRFIP1, HOXB3, CHD6, C3orf38, BNC1, GALNT7, SLC1A3, CFL2, FAM63B, ZNF71, **CLDN1**, HECTD2, KHDRBS2 PPT1, SERPINE2, UBA6, MTF2, C7orf58, SRGN, **SCARB1**, OSBPL11, OLA1, BTF3L4, KRIT1, CAMSAP1, C13orf27, BRMS1L, NONO, DARS, TPH1, … |
| miR-885 | GALNT3, ZNF367, OXR1, CTNNB1, SLC11A2, FAM108C2, ARPP21, UPF2, MAP4K3, ELF4G2, CPEB3, BPY2, **CLDN1**, FUBP3, LOC283508, TTC30A, FRG2, MEX3B, CNTN5, TBCC, B2GALTL, **SCARB1**, LYPLA1, ING3, NEUROD1, LCP1, SMARCE1, MEX3D, LOC100132169, FAM98A, IMPACT, KPNA4, **EGFR**, FAM81A, ELAVL4, TSC2D2, KIF21A, ASPN, UBTD2, NUDCD2, TSPAN13, C9orf3, KIAA1598, MAN1C2 ,JAZF1, CEBPD, LACTB, ME1, |
| miR-1271 | LRCH2, RFPD1, RFPD2, SLC1A1, ADCY6, JMJD1C, COL25A1, FLJ43390, NOVA1, CYYR1, AK3, LPPR4, EOMES, RDH11, RPAP3, DGKH, SOX5, ADK, PTPMT1, MSN, MTRR, OXGR1, LIPG, CAMK2N1, FOXF2, ZFP36L1, HERC2P2, MTSS1, NUS1, HTRA4, ITPR1, NTN4, **CLDN1**, **SCARB1**, SPIN1, RAPGEF4, SDC2, ZNF175, MYRIP, LRRC7, UNC13C, PROK2, NEUROD4, ARHGAP6, CONT6L, SLC39A1, STK17A, C14orf169 FHL1, … |
| Down-regulated miRNAs | Putative target |
| miR-27a | NOVA1, AKIRIN1, HDHD2, PLK2, SBF2, USP42, FBXW7, CDH11, SNAP25, ALCAM, ZDHHC17, RNMT, DUT, ADAM9, MBTD1, EYA4, SNRNP27, CACNA2D3, PLCH1, KRTAP13-2, GCC2, RREB1, PXDN, GALNT7, KIAA0146, YWHAQ, KDM3A, **LDLR**, NRK, RFPD5, RGPD8, **EGFR**, GRIA4, PAX9, ST6GALNAC3, RGPD6, SFXN2, RPS6KA5, MIER3, RGPD4, ANKRD36B, SLC22A25, ARX, MAP2K4, PDE7B, ASPH, NUP133, CARD8, … |
| miR-99a | WNK3, TMPRSS13, TASP1, SMARCA5, EPDR1, AP1AR, KBTBD8, ZC3HAV1, MTOR, EIF2C2, HS3ST2B1, HS3ST2, RNF2, FZD8, RAP1B, ZNRF2, TTC39A, ZNF19, CTDSPL, CASC4, OSBP, ST7OT4, C2orf67, TCF7, PMPCB, GIMAP8, BAT2L2, JARID2, PRKCI, THAP2, ZZEF1, **LDLR**, VNN1, KIAA1715, RAVER2, TARDBP, C9orf123, RNASEH1, ARSJ, CDC25A, TNPO1, … |
